# Molecular Study of* Acinetobacter baumannii* Isolates for Metallo-*β*-Lactamases and Extended-Spectrum-*β*-Lactamases Genes in Intensive Care Unit, Mansoura University Hospital, Egypt

**DOI:** 10.1155/2017/3925868

**Published:** 2017-05-08

**Authors:** Nashwa M. Alkasaby, Maysaa El Sayed Zaki

**Affiliations:** ^1^Medical Microbiology and Immunology Department, Faculty of Medicine, Mansoura University, Mansoura, Egypt; ^2^Clinical Pathology Department, Faculty of Medicine, Mansoura University, Mansoura, Egypt

## Abstract

*Acinetobacter baumannii (A. baumannii)* has been known as a causative pathogen of hospital acquired infections. The aim of this study is to examine the presence of* A. baumannii* among clinical isolates from intensive care unit (ICU) in Mansoura University Hospital (MUH), its antibiotic resistance pattern, and prevalence of antibiotic resistance genes metallo-*β*-lactamases (MBLs) and extended-spectrum-*β*-lactamases (ESBLs) among* A. baumannii* isolates.* A. baumannii* was identified by colony morphology, API 20E, and confirmed by detecting the bla OXA-51-like carbapenemase gene by PCR. Phenotypic expression of MBLs resistance was demonstrated by Combined Disk Test (CDT) in 273 isolates (97.5%) and of ESBLs was demonstrated by double disc synergy method (DDST) in 6 isolates (2.1%). MBLs genes were positive in 266 isolates (95%) and ESBLs genes were positive in 8 isolates (2.9%). The most frequent genes of MBLs studied genes were IMP (95.7%) followed by SIM and GIM (47.1% and 42.9%; resp.). For ESBL genes, the most frequent gene was TEM (2.9%). From this study, we conclude that multidrug resistant (MDR)* A. baumannii* with MBLs activity was the most common isolate. Careful monitoring for the presence of MDR* A. baumannii* among hospitalized patients is recommended to avoid wide dissemination of antibiotic resistance.

## 1. Introduction

*Acinetobacter baumannii (A. baumannii)* is a Gram-negative coccobacilli nonglucose fermentative opportunistic pathogen [1].* A. baumannii* is one of the most important nosocomial pathogens because of its longevity in the hospital environment and ability to resist various antimicrobial agents and to colonize susceptible patients treated with broad-spectrum antibiotic [2]. It has been related to various sites of infections like urinary tract, skin and soft tissue infections, pneumonia, and bacteremia especially in immunosuppressed patients [3–5].

Multidrug resistant* A. baumannii* (MDR* A. baumannii*), defined as an* A. baumannii* strain resistant to at least three different groups, penicillins and cephalosporins (including inhibitor combinations), fluoroquinolones, and aminoglycosides, has emerged and has been reported worldwide to significantly increase the morbidity, mortality, and cost of treatment [6]. The incidence of MDR* A. baumannii* is increasing worldwide, including Europe, North America, Latin America, and Asia [7].

*Acinetobacter *spp. exhibit multidrug resistance through production of *β*-lactamases, alterations in outer membrane proteins (OMPs) and penicillin-binding proteins (PBPs), and increased activity of efflux pumps [8]. Resistance to *β*-lactams appears to be primarily caused by production of *β*-lactamases which include extended-spectrum-*β*-lactamases (ESBLs), metallo-*β*-lactamases (MBLs), and oxacillinases [3].

MBLs are classes of powerful enzymes called carbapenemases responsible for antibiotic resistance. Four groups of these enzymes have been described in* A. baumannii*, including IMP-like, SIM-1, NDM-type, and VIM-like carbapenemases. MBLs-encoding genes are located on integrons that can be transmitted from one bacterial species to another [9, 10]. Extended-spectrum-*β*-lactamases (ESBLs) are encoded by TEM-type, SHV-type, and CTX-M-type genes; they are resistance to penicillins and third-generation cephalosporins [11].

The drug of choice to treat nosocomial infection caused by MDR* A. baumannii *is the carbapenems. However, there is an increasing rate of carbapenem-resistant* A. baumannii *around the world [12].

There are limited data about the prevalence of* A. baumannii* among clinical isolates from Egypt and its pattern of antibiotics resistance. Therefore, we have performed this study to examine the presence of* A. baumannii* among clinical isolate in intensive care units (ICU) from one Egyptian tertiary care hospital, its antibiotic resistance pattern, and the prevalence of antibiotic resistance genes MBLs and ESBLs.

## 2. Material and Method

This cross-sectional study was carried out in the main microbiology laboratory at Mansoura University Hospital, Egypt, between January 2014 and January 2016. The study was approved by Mansoura Faculty of Medicine ethical committee. Clinical samples were obtained from patients admitted to ICU including blood, urine, sputum endotracheal secretion, wound swabs, pus, and CSF.

### 2.1. Culture and Identification

The specimens were inoculated on blood agar, chocolate agar, and MacConkey agar medium and incubated for 24 h at 37C. Culture growth was identified to be* A. baumannii* growth by colony morphology and API 20 E confirmed by detecting bla OXA-51 carbapenemase gene using PCR [13]. Antibiotics susceptibility tests were done followed by multiplex PCR for detection of MBLs genes and ESBLs genes.

### 2.2. Antimicrobial Susceptibility

#### 2.2.1. Standard Disc Diffusion Technique

*A. baumannii *isolates were tested for the susceptibility to ceftazidime (30 *μ*g), cefepime (30 *μ*g), imipenem (10 *μ*g), meropenem (10 *μ*g), gentamicin (10 *μ*g), amikacin (30 *μ*g), ciprofloxacin (5 *μ*g), levofloxacin (5 *μ*g), sulfamethoxazole/trimethoprim (1.25/23.75 *μ*g), and piperacillin/tazobactam (100 *μ*g/10 *μ*g).

These analyses were performed by standard disc diffusion technique and interpreted according to the Clinical and Laboratory Standards Institute (CLSI) guidelines [16].

#### 2.2.2. *E*-Test (MIC Determination)

The *E*-test was used to determine antimicrobial susceptibility and the minimum inhibitory concentration (MIC) for tigecycline and colistin susceptibility according to the CLSI guidelines. Minimum inhibitory concentration (MIC) breakpoints for colistin were resistance, ≥4 mg/L, and susceptibility, ≤2 mg/L, and for tigecycline were resistance, ≥8 mg/L, and susceptibility, ≤2 mg/L.* E. coli *ATCC 35218 and* Pseudomonas aeruginosa *ATCC 27853 were used in all tests as control strain [16].

### 2.3. Phenotypic Detection of MBLs Activity

#### 2.3.1. Imipenem-EDTA Combined Disc Test (CDT)

Isolates resistant to imipenem were selected for detection of MBLs enzymes by imipenem-EDTA combined disk method. The test isolates (opacity adjusted to 0.5 McFarland opacity standard) were cultured on Mueller-Hinton agar plate as recommended by CLSI. After drying, two 10 *μ*g imipenem discs were placed on the culture plate with 20 mm distance from center to center of the discs. 10 *μ*l of 0.5 M EDTA was added to one of the imipenem discs and incubated overnight. Isolates showing ≥7 mm increase in the inhibition zone size of imipenem-EDTA disc compared to the imipenem disc alone were considered as MBLs producers [17].

### 2.4. Phenotypic Identification of ESBL Producing Isolates

#### 2.4.1. Double Disc Synergy Test (DDST)

Isolates resistant to third-generation cephalosporins were tested for ESBLs production by CLSI double disc synergy method (DDST). Antibiotic disks containing ceftazidime (30 *μ*g), cefotaxime (30 *μ*g), ceftazidime (30 *μ*g) + clavulanic acid (10 *μ*g), and cefotaxime (30 *μ*g) + clavulanic (10 *μ*g) were used.

Pairs of disks (ceftazidime with ceftazidime/clavulanic acid and cefotaxime with cefotaxime/clavulanic) were placed on Muller-Hinton agar medium with 20 mm space between them.

According to the CLSI criteria and manufacturer instruction, the ≥5 mm inhibition zone of growth in ceftazidime/clavulanic acid and cefotaxime/clavulanic compared to ceftazidime and cefotaxime was regarded as an ESBLs producing isolate [18].

### 2.5. Multiplex PCR

#### 2.5.1. DNA Extraction

Pure colonies of* A. baumannii* were used for DNA extraction by Qiagen kit according to the manufacturer recommendations and kept at −70°C till amplification reactions.

#### 2.5.2. Polymerase Chain Reaction (PCR)

*(a) PCR for the Detection of blaOXA-51-Like Gene.* Polymerase chain reaction (PCR) assay was performed for the detection of* bla*OXA-51-like gene, a 353-base-pair (bp) amplicon, as an internal gene for molecular confirmation of* A. baumannii *isolates at the species level primer sequence: F/5′-TAA TGC TTT GAT CGG CCT TG-3′, R/5′-TGG ATT GCA CTT CAT CTT GG-3′ [13].

*(b) Multiplex PCR.* Multiplex PCR was used to screen for the following resistance genes as previously described [14, 15].

Metallo-*β*-lactamases are blaIMP, blaVIM, blaGIM, bla SPM, and blaSIM-1 and class A beta-lactamases are blaTEM, blaSHV, and blaCTX-M. Primers used in this study are listed in Table 1. For the amplification procedures we used 3 microns of the extracted DNA applied over 25 microns of ready to use master mix with 0.2 microns of Taq polymerase 5 U/*μ*l (Qiagen) with l *μ*l of each primer (10 pmol/ml) from each reverse and forward primer.

For PCR detection of amplified genes, agarose gel electrophoresis of the amplified DNA product with 100 bp size marker was carried out in a 2% agarose gel for 2 h at 80 V and stained with ethidium bromide and visualized under UV transillumination [19].

## 3. Results

This study included 280 of* A. baumannii* isolated from different clinical samples from intensive care unit, Mansoura University Hospitals during 24 months.* A. baumannii *was confirmed by detecting bla OXA-51 gene in all isolates.* A. baumannii* isolates were most frequently recovered from endotracheal secretion (39.3%) followed by sputum (23.8%), pus/wound swab (14.3%), blood (11.8%), urine (10.7%), and cerebrospinal fluid (0.4%), Table 2.

*A. baumannii* isolates were highly resistant to imipenem, meropenem, ceftazidime, and cefepime with resistance rate of 95.7%, 95.7%, 96.4%, and 97.1%, respectively, Table 3. Also, the isolates showed high frequency of resistance to ciprofloxacin, gentamicin, sulfamethoxazole/trimethoprim, piperacillin/tazobactam, and amikacin (93%, 85%, 89%, and 95%, resp.).

Regarding colistin and tigecycline sensitivity, lower resistance was reported for these antibiotics (3% and 1.5%, resp.).

The phenotypic expression of MBLs resistance was demonstrated by CDT in 273 isolates (97.5%) and of ESBLs was demonstrated in 6 isolates by DDST (2.1%).

Genotypes detection for the studied genes for MBLs was positive in 266 isolates (95%) and for ESBLs was positive in 8 isolates (2.9%). Totally 280* A. baumannii* isolates have been examined for 5 MBLs and 3 ESBLs encoding genes. For MBLs genes, the most frequent genes were IMP (95.7%), followed by SIM and GIM (47.1% and 42.9%, resp.), Table 4. The most frequent genes from ESBL studied genes were TEM (2.9%), SHF (2.1%), and CTX-M in 1.8%, Table 5. Some of the isolates had more than one gene.

## 4. Discussion

Multidrug resistant (MDR)* A. baumannii* that are present in the ICU and in the hospital environment represent therapeutic problem because of resistance to most antimicrobial agents [1].

In the present study,* A. baumannii* was isolated most commonly from the respiratory tract specimen followed by wounds and blood. This is consistent with data presented by other studies where the major source of* A. baumannii* isolates was respiratory specimens followed by wounds [20, 21].

Antibiotic resistance rate was high for most antibiotic except for colistin and tigecycline. The resistance of* A. baumannii* isolates to cephalosporins in the present study was in accordance with another study conducted by Safari et al., 2013 [5]. Ciprofloxacin resistance rate (92.9%) was similar to studies conducted in Egypt and India which showed resistance rate of 85% and 99%, respectively [22, 23].

Regarding aminoglycoside, resistant rate is 90%; this is in accordance with other studies [24]. This finding is a warrant index to the possibility of coexistence of genes responsible for aminoglycoside and carbapenem resistance on the same genetic elements that may explain high resistance level to the tested aminoglycosides in the present study [7].

Carbapenems are generally the last resort in the treatment of life-threatening infections caused by MDR* A. baumannii.*

*A. baumannii* strains isolated in the present study had high frequency resistance to imipenem, meropenem. The presence of high resistance to carbapenem has been reported previously in Egypt [22, 25, 26] and Turkey [20]. This finding can be explained by inadequate adherence to infection control guideline in addition to inappropriate use of carbapenem [26].

The preliminary resistance pattern of the isolated* A. baumannii *directs the attention to the presence of MBLs that was proven by phenotypic study by Combined Disk Test (CDT) for MBLs with a high frequency of MBLs gene (95%). Previous reports stated that MBLs production among* A. baumannii* range from 49% up to 99% [27–29].

In the present study the presence of phenotypic ESBLs was detected in 2.1% of the isolates. On the contrary, higher results were reported in previous studies for ESBL producers bacteria with range from 20% up to 85% [29–31].

Treatment of patients infected with MBLs producers is challenging due to the currently limited options. Colistin and tigecycline are currently the only treatment choice for these MDR* A. baumannii*. Fortunately our finding in the present study showed that resistance rate to colistin and tigecycline is low (3% and 1.5%) and was in accordance with other studies [32, 33].

In our study, all isolated* A. baumannii *strains carried bla OX-51 gene and were confirmed as* A. baumannii*. This gene is considered as a natural component of the species and may be associated with resistance to carbapenems [34].

Genotypes detection of the studied genes for MBLs was positive in 266 isolates (95%) and for ESBLs was positive in 8 isolates (2.9%). Among MBLs, IMP gene was the most detected gene as reported previously in other studies [35, 36]. IPM and VIM genes have been reported from different countries with different prevalence rate [37–39].

On the contrary, IPM and VIM genes were not detected in study conducted in Egypt by Fatouh and El-din 2014 [40]. SPM gene was not detected in the present study; this is in accordance with another study conducted in Iran by Moghadam et al. 2016 [41]. Among ESBLs, TEM gene was positive in 2.9% of* A. baumannii* isolates; higher result was reported (13.15%) by Rezaee et al., 2013 [35].

## 5. Conclusion

MDR* A. baumannii* isolates with metallo-*β*-lactamases activities represent therapeutic problem in ICU.

The main gene encoding for these metallo-*β*-lactamases in our study was IMP. MDR* A. baumannii* showed high resistance rate to most of the available antimicrobial agents, except for tigecycline and colistin.

Careful monitoring for the presence of MDR* A. baumannii *among hospitalized patients is recommended to avoid wide dissemination of antibiotics resistance and to limit the indiscriminative use of cephalosporins and carbapenems in the hospital. Antibiotics Stewardship Program and infection control measures environment should be applied to minimize the emergence of multiple drug resistant bacteria.

Physician should treat infection and not colonization based on clinical signs and laboratory parameters indicating systemic inflammatory response/sepsis.

## Figures and Tables

**Table 1:** Primers used for PCR amplification of the studied genes.

Gene	Sequence	bp	Reference
CTX-M	F: ATGTGCAGTACCAGTAAGCGTCATGGC R: TGGGTAAAATATGTCACCAGAACCAG	593	[14]
SHV	F: ATGCGTTATATTCGCCTGT R: TGCTTTGTTATTCGGGCCAA	747	[14]
TEM	F: CGC CGC ATA CAC TAT TCT CAG AAT GA R: ACG CTC ACC GGC TCC AGA TTT AT	445	[14]
VIM	F: GATGGTGTTTGGTCGCATA R: CGA ATGCGCAGCACCAG	390	[15]
IMP	F: GGAATAGAGTGGCTTAAYTCTC R: CCA AACYACTASGTTATCT	188	[15]
SPM-1	F: AAAATCTGGGTACGCAAACG R: ACATTATCCGCTGGAACAGG	271	[15]
SIM-1	F: TAC AAGGGATTCGGCATCG R: TAATGGCCTGTTCCCATGTG	570	[15]
GIM-1	F: TCG ACACACCTTGGTCTGAA R: TCG ACACACCTTGGTCTGAA	477	[15]

**Table 2:** *Acinetobacter baumannii* distribution according to the site of infection.

Sample	Number	%
Endotracheal secretion	110	39.3
Sputum	66	23.8
Pus/wound swab	40	14.3
Blood	33	11.8
Urine	30	10.7
CSF	1	0.4

**Table 3:** Antibiotics resistant patterns in *Acinetobacter baumannii* isolates.

Antibiotics	Number	%
Ceftazidime	270	96.4
Cefepime	272	97.1
Imipenem	268	95.7
Meropenem	268	95.7
Ciprofloxacin	260	92.9
Gentamicin	260	92.9
Sulfamethoxazole/trimethoprim	218	77.9
Piperacillin/tazobactam	250	89.2
Amikacin	250	89.2
Colistin	9	3.2
Tigecycline	5	1.8

**Table 4:** MBLs genes detected in *Acinetobacter baumannii* isolates.

Genes	Number	%
IMP	268	95.7
SIM	132	47.1
GIM	120	42.9
VIM	0	0
SPM	0	0

**Table 5:** ESBLs genes detected in *Acinetobacter baumannii* isolates.

Genes	Number	%
TEM	8	2.9
SHV	6	2.1
CTX-M	5	1.8
